# High turnover of faecal microbiome from algal feedstock experimental manipulations in the Pacific oyster (*Crassostrea gigas*)

**DOI:** 10.1111/1751-7915.13277

**Published:** 2018-05-10

**Authors:** Ariel Levi Simons, Nathan Churches, Sergey Nuzhdin

**Affiliations:** ^1^ Marine and Environmental Biology University of Southern California Los Angeles CA USA; ^2^ Molecular and Computation Biology University of Southern California Dana and David Dornsife College of Letters Arts and Sciences Los Angeles CA USA

## Abstract

The composition of digestive microbiomes is known to be a significant factor in the health of a variety of hosts, including animal livestock. Therefore, it is important to ascertain how readily the microbiome can be significantly altered. To this end, the role of changing diet on the digestive microbiome of the Pacific oyster (*Crassostrea gigas)* was assessed via weekly faecal sampling. Over the course of 12 weeks, isolated individual oysters were fed either a control diet of *Tetraselmis* algae (*Tet*) or a treatment diet which shifted in composition every 4 weeks. Weekly faecal samples from all oysters were taken to characterize their digestive bacterial microbiota. Concurrent weekly sampling of the algal feed cultures was performed to assess the effect of algal microbiomes, independent of the algal type, on the microbiomes observed in the oyster samples. Changing the algal feed was found to be significantly associated with changes in the faecal microbiome over a timescale of weeks between control and treatment groups. No significant differences between individual microbiomes were found within control and treatment groups. This suggests the digestive microbiome of the Pacific oyster can be quickly and reproducibly manipulated.

## Introduction

The microbiome has increasingly been identified as playing a critical role in a host's various physiological functions, ranging from infection response (Wen *et al*., 2008) to digestion (Bahrndorff *et al*., 2016). Gaining a better understanding of host–microbiome interactions, then, is of particular interest in a variety of fields ranging from human health (Belkaid and Hand, 2014) to food management techniques (García‐Orenes *et al*., 2013). Modern agriculture has benefited directly from associative phenotype‐microbiome studies. For example, soil microbial communities, an analog for animal microbiomes, have been shown to (i) affect the efficiency of plant nutrient uptake via solubilization of minerals, (ii) guard plants against pathogenic organisms and (iii) regulate growth via synthesis of plant hormones (see review: Hayat *et al*., 2010). This work has led to the commonplace use of microbial inoculation and ‘microbe‐encouraging’ soil mixtures in terrestrial commercial and hobbyist plant cropping. For livestock farming, microbiome studies are becoming more common as the link between products, such as meat and milk, and associated microbiomes becomes clearer (Mackie, 2002; Sommer and Backhed, 2013). For the dairy cow, microbiome studies have revealed that each separate stomach contains distinct microbiomic profiles, which are associated with differential genetic regulation in each organ (Mao *et al*., 2015). As a whole, the cow microbiome has been associated with phenotypes of commercial interest such as disease (Nakamura *et al*., 2017) and food conversion efficiency (e.g. Myer *et al*., 2017). Recent work on humans also suggests a degree of vertical inheritance for the gut microbiome (Bäckhed *et al*., 2015). Increased understanding of host–microbiome phenotypic associations will likely continue to play an ever more important role in food management and breeding strategies, as well as human medicine.

This is especially true in commercial aquaculture, where evidence suggests that host–microbiome interactions could have a role both in the management of bacterial pathogens (Tan *et al*., 2016), as well as improving host growth rates (Douillet and Langdon, 1994; Martínez Cruz *et al*., 2012). Within aquaculture, a better understanding of these interactions would be of particular interest to the cultivation of Pacific oysters [*Crassostrea gigas, (Cgi)*], a significant portion of a $19 billion global aquaculture industry (FAO, 2016). Although they are becoming more common, genetic improvement strategies for oysters are difficult processes due to their multiyear life cycles, and the inherent difficulty in retaining pedigreed individuals in oceanic environments. Even a moderate increase of 5% in growth rate would allow farmers to reduce time to market by between 27 and 54 days (FAO, 2005). Furthermore, genomic resources are still somewhat lacking for *Cgi*. While its genome has been recently sequenced (Zhang *et al*., 2012), and quantitative trait loci analysis has demonstrated a genetic component to desirable traits such as growth rate (Guo *et al*., 2011), it has still proven difficult to apply this knowledge towards improvements in oyster crop via selective breeding (Dégremont *et al*., 2015). A better understanding of how to improve commercial phenotypes of cultured oysters through influencing their microbiota might provide a shorter route to crop improvement for oysters than genomic approaches. For example, improvements in larval growth rates of approximately 20% were reported through the addition of a probiotic bacterial strain to the feed of *Cgi* (Douillet and Langdon, 1994).

Human health is also adversely impacted by a general lack of knowledge of bivalve microbiomes and associated physiological phenomenon because these animals are frequently consumed raw and in their entirety. Consumers therefore ingest the whole of the bacterial community also, which may transfer highly toxic pathogenic bacteria (Givens *et al*., 2014). This can lead to deadly human diseases such as vibriosis, which is fatal in 15–30% of cases (U.S. Food and Drug Administration, 2003). On an ecological scale, the monitoring of bivalve microbiomes could be used as an early warning system for the onset of harmful algal blooms (McPartlin *et al*., 2016), which may also cause lethal human health issues such as paralytic shellfish poisoning (Hurley *et al*., 2014). Many rural communities depend largely on wild‐harvested bivalves as a source of protein, and the only way to completely avoid the risk of PSP is to eliminate this food staple completely (Paralytic Shellfish Poisoning Fact Sheet, 2002). Addressing the question of how readily *Cgi* can accumulate pathogens from their environment, as well as assessing the efficacy of current pre‐market depuration methods for commercial *Cgi* crops, would be quite useful to the field of public safety.

Taken together, it is clear that commercial aquaculture and human food safety management would benefit from a deeper understanding of how readily the microbiome, and in particular digestion associated microbiome, of culvitated *Cgi* can be altered. However, the literature concerning bivalve‐associated microbiota is still in its infancy, and the transferability of techniques and approaches between species has not been established. Previous work with *Crassostrea virginica* (King *et al*., 2012) has demonstrated a differentiation in the gut microbiome of populations from different geographic localities, most likely due to regionally distinct marine bacterial communities. Similar results have been shown with *Cgi* and, using a combination of antibiotics and transplantation, significant shifts in the composition of the gut microbiome have been observed over the course of a week (Lokmer *et al*., 2016). This study aims to establish the first associations between diet and corresponding microbiome profiles in *Cgi*. Here, by controlling environmental parameters and adjusting diets between conspecifics of *Cgi*, dietary variance was shown to correspondingly affect the digestive microbiome. As our group has used the same laboratory set‐up and similar feeding schedule to do related work with other bivalves, such as the mussel *Mytlius galloprovincialis*, there is the potential to apply this study's methods to other farmed shellfish species.

## Results

### Sequencing and OTU Visualization

**Figure 1:**
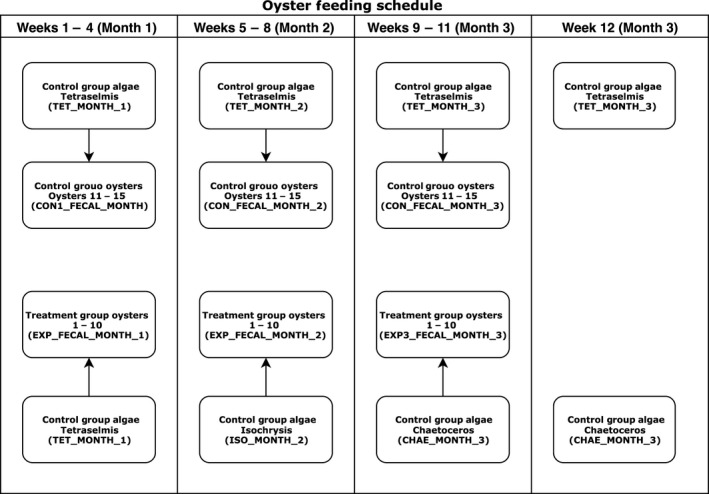
Experimental Sampling Schema: Oyster feeding and sampling schedule with naming schema. The month in this experiment refers to the particular 4 week window of time for a given algal culture used in feeding the treatment group of oysters. Faecal and algal samples were collected weekly. Direct gut extraction samples were collected during week 12, but not used in analysis due to low read depth. No faecal samples were collected during week 12. Further details described in the materials and methods section.

In total, approximately 3.81 million high‐quality paired‐end read sequences, clustered at 97% similarity into 4009 Operational Taxonomic Units (OTUs), were identified across the 128 samples successfully sequenced in this study (see Fig. 1 for sampling scheme). The distribution of sequences assigned per OTU is highly skewed with approximately 50% of all counted sequences found in the 11 most abundant OTUs, and 90% of all sequences found within the most abundant 144 OTUs.

**Figure 2:**
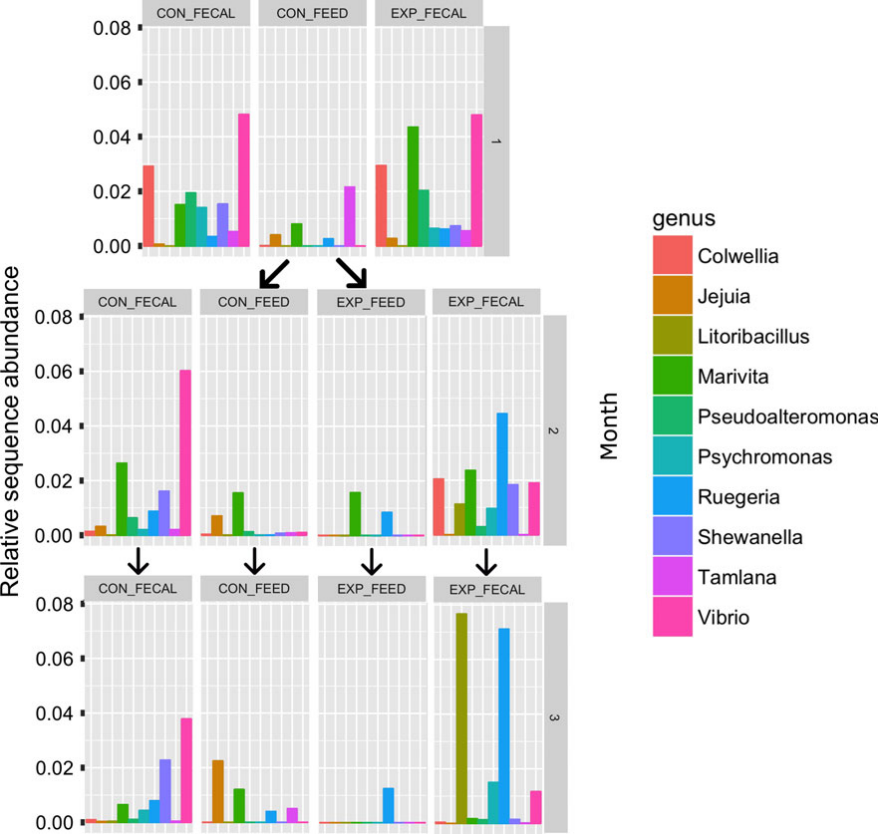
Bacterial Community Composition Changes: The relative abundances of the 10 most prevalent bacterial genera found in all algal and faecal samples. Note that the control feed, CON_FEED, represent the *Tet* feedstock and is shared between control and treatment groups during the first month. The treatment feed, EXP_FEED, represents the *Iso* feedstock during month 2 and *Chae* feedstock during month 3. CON_FECAL represents faecal samples from the control group of oysters, which are only feed Tet, and EXP_FECAL represents faecal samples from the oysters receiving the treatment feed.

Of the 128 samples analysed in this study, 17 comprise the bacterial communities found in the algal feedstock, 103 comprise the bacterial communities found in the oyster faecal pellets and eight comprise the samples directly extracted from the stomach of the oysters during the final week of sampling. While the read depth for the samples directly extracted from the gut was too low to use for statistical analysis [under 1000 reads per sample (See Table S1)], some individual taxa could be identified. For the oysters feeding on *Tet* at the time of extraction, their stomach communities were dominated by *Deinococcus*,* Shewanella*,* Marivita* and *Vibrio*. For those feeding on *Chae* at the time of extraction, their gut communities were dominated by *Albimonas* and *Ruegeria*. Figure 2 shows relative sequence abundance of the top 10 most represented bacterial genera between control and experimental treatment groups in the 120 remaining samples after filtration. It is visually clear that bacterial community composition diverges as a function of both algal type and treatment group (control vs. experimental), suggesting a digestive microbiome that is dynamically responsive to feed type.

### MDS Plots and β‐diversity

Samples were then analysed using techniques described in Lokmer *et al*. (2016). The significance of the experimental factors explaining observed measures of β‐diversity was determined using a permutational multivariate analysis of variance (Permanova) on the Bray–Curtis dissimilarity values between samples. Both the weighted Unifrac distance and Bray–Curtis dissimilarities between samples yielded similar results (Figs S1–S10). Visualizing these differences was done using multidimensional scaling (MDS) plots, with the two axes describing the largest amount of total variation in β‐diversity, to reduce the dimensionality of the microbiome data.

### Algal microbiomes

**Figure 3:**
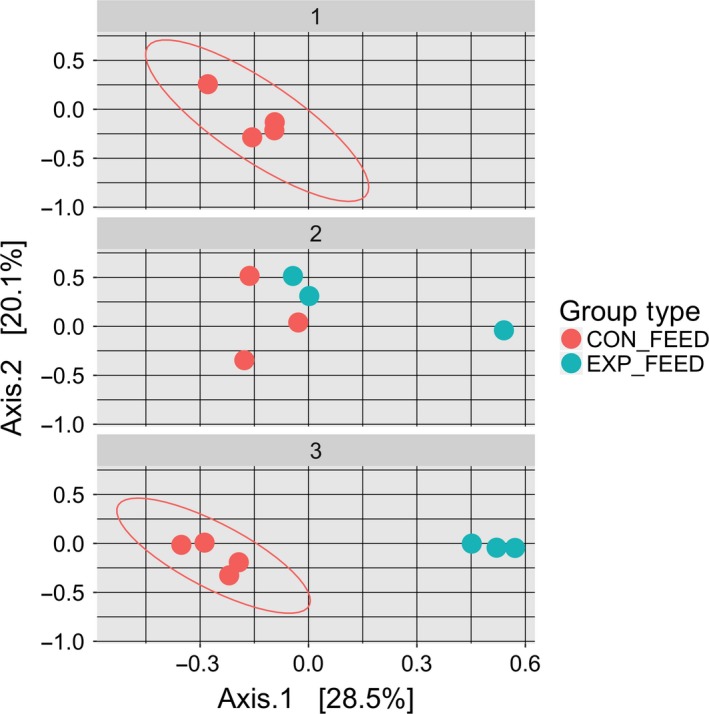
Differentiation in Algal Associated Micobiomes: MDS plot, using the Bray–Curtis dissimilarity between algal samples during each month of the study. CON_FEED represents the control *Tet* feedstock, which is shared between control and treatment groups during the first month. The treatment feed, EXP_FEED, represents the *Iso* feedstock during month 2 and *Chae* feedstock during month 3. Note that groups with less than four samples have too few points for a unique ellipse to be drawn using the stat_ellipse function in Phyloseq.

It was observed that each algal culture had distinct associated bacterial communities. MDS plots show that for the control feed, which consisted of a continuous culture of *Tet* throughout the 3 month experiment, the bacterial communities remained stable, whereas experimental feed bacterial communities changed with algae culture type, *Isochrysis* spp. (*Iso*) or *Chaetoceros* spp. (*Chae*) (Figs S2 and S7). When comparing control feeds to experimental feeds within months, algae type was a significant factor in explaining the Bray–Curtis dissimilarity between each algal‐associated microbiome sample [*F*(2,14) = 3.0974, *P* = 5e‐4] for the duration of this study (Fig. 3). While changes in the algal microbiomes were easy to observe in all algal samples, even in control feedstocks on a monthly basis, weekly sampling time was found not to play a significant role in determining the composition of each algal microbiome [*F*(1,15)  = 1.1433, *P* = 0.3246]. This supports the idea that algae communities are relatively stable within feed types, but distinct between them. Time was not a significant factor in explaining the algal *Tet* microbiome at both the weekly [*F*(1,9) = 1.3948, *P* = 0.2243] and monthly timescales [*F*(1,9) = 1.663, *P* = 0.1099], which indicates a stable algal microbiome composition over time.

### Oyster faecal microbiomes

**Figure 4:**
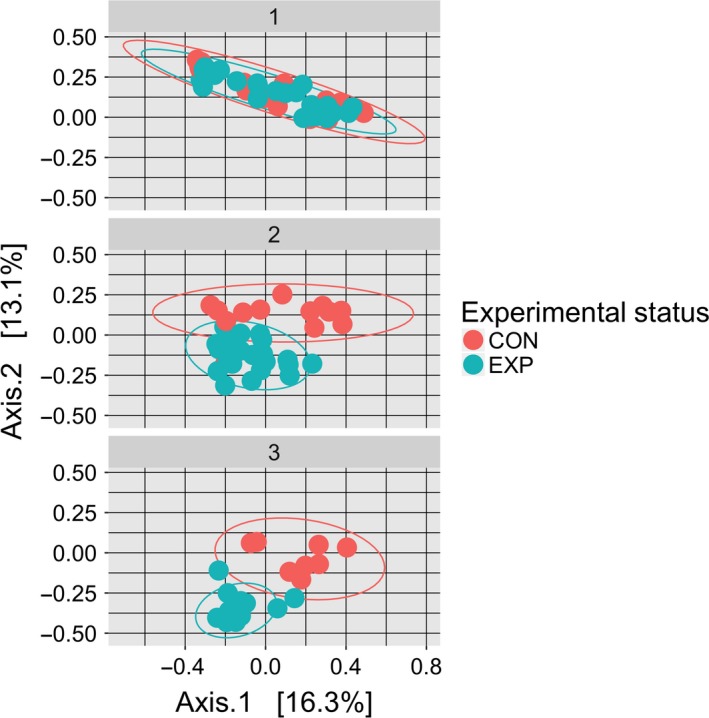
Divergence in Digestive Microbiomes by Experimental Status: MDS plot, using the Bray–Curtis dissimilarity between faecal samples during each month of the study. CON represents faecal samples from oysters in the control group, which are always feed Tet, and EXP represents faecal samples from oysters in the treatment group. Both control and treatment groups are fed *Tet* during the first month. In months 2 and 3 the EXP group is feed *Iso* and *Chae* respectively.

The 103 faecal samples observed were significantly differentiated based on the type of feed received [*F*(2,99) = 9.9143, *P* < 1e‐4]. When comparing the control group to experimental group in month 2 (*Tet* vs. *Iso*) and month 3 (*Tet* vs. *Chae*), significant differences were found in faecal sample community composition [Fig. 4. *F*(1,35) = 5.9068, *P* < 1e‐4, and *F*(1,29) = 7.5761, *P* < 1e‐4, respectively]. For the faecal samples taken from oysters in the control group, the composition of each sample's microbiome did not remain stable on a weekly timescale [*F*(1,37) = 2.7147, *P* = 0.005999], or on a monthly timescale [*F*(1,37) = 3.3479, *P* = 0.0014]. However, replicates were not reported as a significant factor in determining bacterial community composition within the faecal control [*F*(1,37) = 1.2451, *P* = 0.1242] or faecal treatment [*F*(1,71) = 1.0083, *P* = 0.447] groups.

Next, the faecal microbiomes from the control and treatment groups of oysters were compared as feedstocks were changed. During the first month of this experiment, both the control and treatment groups of oysters received identical *Tet* feed. It was found that oysters raised in identical conditions on identical diets will tend to cultivate highly similar bacterial communities as experimental status was found not to be a significant factor in differentiating faecal microbiomes [Fig. 4; *F*(1,41) = 1.0903, *P* = 0.3208]. During the second month, when the treatment group received *Iso* algae, the faecal microbiomes diverged with experimental status becoming a significant factor in explaining differences in faecal microbiome compositions [Fig. 4; *F*(1,35) = 5.9068, *P* < 1e‐4]. During the third month, for faecal samples obtained during weeks 9 through 11, experimental status becomes an even more significant factor in describing differences between faecal microbiomes [Fig. 4; *F*(1,20) = 8.0843, *P* < 1e‐4].

### Temporal variability in microbiomes

The results for both algal and faecal microbiome comparisons are in general agreement with observations made of changes observed in the relative abundance of the most common sample taxa over time (Fig. 2), as well as an extended Local Similarity Analysis (eLSA) of patterns of co‐occurrence between common taxa across samples (See Table S2). The faecal and algal microbiomes remained significantly distinct throughout the entire course of the study for the control group [*F*(1,48) = 8.7385, *P* < 1e‐4; See Figs S4 and S5, S9 and S10]. For the treatment groups, there was significant differentiation between the faecal and algal microbiomes [*F*(1,71) = 5.0757, *P* < 1e‐4] for the duration of the study, similar to the control group. The monthly timescale was also a significant factor in differentiating both algal [*F*(1,8) = 3.4973, *P* = 0.0043] and faecal microbiomes [*F*(1,61) = 10.386, *P* < 1e‐4].

## Discussion

### Algal microbiomes

How strongly associated are the algal microbiomes to a particular algal culture? While the water used in culturing algae was taken from a water supply passed through a 1 μm filter and a UV treatment, the presence of algal microbiomes illustrates that these are not axenic cultures. Indeed, viability would be low, or impossible, in a completely axenic microalgae culture, as many microalgae species are auxotrophic for bacterially generated micronutrients (Kazamia *et al*., 2012). Auxotrophic associations are known to exist between the algae and their associated bacterial communities frequently found in this study, such as those between *Ruegeria* and *Tet* or *Iso* (Arora *et al*., 2012) or *Maritivita* and *Chae* (Kimura and Tomaru, 2014; Cruz‐López and Maske, 2016). Similarly, a number of bacteria found in the algal communities are known consumers of algae, and algal exudates, such as members of *Tamlana* and *Ruegeria* (Arora *et al*., 2012; Chauhan and Saxena, 2016). These relationships are reflected in the composition of our algal culture communities, although underlying factors were not the focus of this study. It should be noted that even within a controlled environment the bacterial communities within the *Tet* culture varied over the course of the study. This could suggest internal community dynamics within the algal, variations in the concentrations of trace elements in the water supply, or both (Harrold *et al*., 2018).

### Oyster faecal microbiomes

In general, the composition of the bacterial communities found in the faecal samples is significantly different, with one trend being a significant rise in the relative abundance of genera such as *Litoribacillus*,* Shewanella* and *Vibrio*. The rise in the relative abundance of these bacterial genera is not unexpected as a number of their member species are known copiotrophs (Goldberg *et al*., 2017; Kim, 2017). These observations fall in line with studies showing the prevalence of the phyla *Proteobacteria*, and in particular members of *Vibrio*, and *Bacteroidetes* in *Cgi* faecal communities (Hernández‐Zárate and Olmos‐Soto, 2006; Fernández *et al*., 2014; Wang *et al*., 2014). These results also suggest that it may be possible to design future experiments to elucidate methodologies for earlier and faster prediction of vibriosis promoting conditions in natural environments.

In observing the most abundant bacterial taxa at monthly intervals (the frequency at which the treatment group algal feed was changed), a number of the patterns were observed with the analysis of the samples’ bacterial community composition. One, the dominant taxa in the faecal microbiomes appear distinct from their corresponding algal microbiomes for both the control and treatment group of oysters. This would suggest a role for the oyster gut environment in shaping a bacterial community to being significantly different from that of its food. This study therefore reflects previously observed differences between the bacterial communities in the local water column and in the guts of both the Pacific and Eastern oysters (King *et al*., 2012; Lokmer *et al*., 2016). Two, the dominant bacterial taxa are distinct between algal cultures. Three, there is a significant divergence observed in the control and treatment faecal groups as the treatment algal feed changes after the first month. This suggests a role for algal feedstock in shaping the digestive bacterial community, and that such shaping can happen on the scale of weeks. Four, the general trend observed in the faecal bacterial communities were that they contained a high relative abundance of copiotrophic genera as compared to the algal bacterial communities, independent of the time or type of algae. For example, the dominant bacteria found in the *Tet* feedstock varies over time, but the faecal communities in the control group oysters fed only *Tet* consistently have a significant abundance of *Vibrio* and *Shewanella*. A consistent shift is also seen in the faecal samples from the treatment group of oysters, which consistently show a rise in the relative abundance of *Vibrio* as compared to the algal feedstock communities.

What remains to be determined is whether the rise in abundance of specific copiotrophic bacteria in faecal samples is primarily determined by the nutritional profile of the algal culture used as feed, or by another factor such as associated digestive bacterial communities. There was observed a repeated rise in the relative abundance of genera such as *Marivita* and *Vibrio* in the oysters only fed *Tet*, even with a varying composition of the *Tet* culture's bacterial community. This points to a role for the algal culture's nutritional profile in shaping the composition of the gut microbiome, which thereby influences the faecal microbiome community. The fact that faecal samples were collected at the same post‐feeding interval, coupled with no significant difference between within each time point's replicate bacterial communities, suggests that faecal bacterial communities are indeed changing as a function of dietary influence. A future avenue for research would be increasing controlled bacterial doses in feed, and observing associated gut and faecal microbiome response.

## Conclusion

In this study, oyster digestive microbiomes were experimentally manipulated via a change in diet, which provides evidence that a stable, rather than varying, diet will tend to yield a more stable digestive microbiome as assessed from faecal samples. For example, the oysters in the control group consistently showed an abundance of *Vibrio* in their faeces, while those in the treatment had shifts in their faecal bacterial communities at the same timescale as changes in their feed. This study illustrates also that changes in diet can yield significant changes in the composition of the digestive microbiome on the scale of weeks. This plasticity suggests that the digestive microbiome of oysters will be able to respond quite rapidly to perturbations such as the introduction of probiotic or pathogenic bacteria. This work additionally suggests that oysters under similar environmental conditions will have faecal microbiomes which respond similarly to shifts in diet. This may be true regardless of genetic background, as oysters in this study were from a semi‐wild cohort found at an aquaculture farm, although it is conceded that the continuity between replicates may be as easily explained by potentially high genetic homogeneity (i.e. low *N*_e_) in bivalves in wild (Hedgecock and Pudovkin, 2011) and aquaculture farm populations (Hedgecock and Sly, 1990). Furthermore, the fact that shifts in faecal microbiomes were similar between replicates indicates that the experimental condition (i.e. control group or experimental group) was the stimulating factor, as opposed to some unknown protocol variable. The individual was not a significant factor in determining the composition of the faecal microbiome for either the control [*F*(1,37) = 1.2451, *P* = 0.1242] or treatment group of oysters [*F*(1,71) = 1.0083, *P* = 0.447] throughout the course of this study. The authors conclude that further work on studying host–microbiome interactions in Pacific oysters could be done with the expectation of a significant degree of reproducibility between individuals, at least for those reared in a similar environment.

## Materials and methods

### Oyster collection

The 15 oysters used in this study were transported as adults from a semi‐enclosed lagoon at the Carlsbad Aquafarm (Carlsbad, CA, USA) to an aquaculture test facility in the Wrigley Marine Sciences Center (WMSC) located near the town of Two Harbors on Catalina island. In the 3 weeks prior to this study, these oysters were held in a common tank and fed a 1:1 cell mix of *Tet* and *Iso* algal cultures at a density of 100 000 cells/ml, on a daily basis.

### Experimental set‐up and sampling

The oysters were separated into two groups and kept on a regular feeding and sampling schedule for the 12 week duration of the study. The control group (*n *=* *5) was only fed *Tet* for the duration of the study. The experimental group (*n *=* *10) was feed *Tet* for the first 4 week period, followed by *Iso* for the second 4 week period and finishing with *Chae* for the final 4 week period. For the duration of the study, all 15 of the oysters were kept in separate 12 litre tanks containing 1 μm filtered seawater and fed algal cultures at a density of 100 000 cells/ml, 12 h per day, three times per week (FAO, 1975). Following each feeding, the water was changed using the 1 μm filtered seawater supply. All of the algal cultures were grown on site at WMSC in 200 litre containers using the same 1 μm and UV filtered seawater supply.

Each week, the faecal pellets from the oysters, as well as the bacteria pelleted from the algal cultures, were collected. A weekly sampling schedule was chosen as both commercial depuration processes (Lee, 2008), and prior work studying the effects of geographic transplantation on *Cgi* microbiota (Wegner *et al*., 2013; Lokmer *et al*., 2016), have demonstrated that significant shifts in *Cgi* gut communities can regularly occur within 3–5 days. To collect faecal pellets, the oysters were removed from their tanks following a water change and placed into 2 litre containers filled with 0.2 μm filtered UV‐sterilized seawater 12 h prior to sampling. The faecal pellets were collected using individual disposable pipettes and then frozen in 1.5 ml collection tubes at −20°C for later DNA extraction. Concurrent to the oyster faecal pellet collection, separate 15 ml conicals of each feedstock were centrifuged at 13 000 *g* for 5 min and the pellet of algae and bacteria was removed with a disposable pipette and frozen in 1.5 ml collection tubes at −20°C for later DNA extraction. On the 12th and final week of the study, the stomach contents of oysters were extracted instead of collection of faecal pellets. To extract the stomach contents, each oyster was shucked, the stomach surface and surrounding tissues rinsed using a 1% bleach solution (Provost *et al*., 2011), and the stomach directly excised and emptied into a 1.5 ml sample collection tube using a sterile razor blade. These samples were then frozen at −20°C for later DNA extraction.

The final number of usable samples consisted of 110 faecal pellet samples, 10 stomach samples and 18 pelleted algal samples. The typical sample volume for the faecal pellets and the pelleted algal cultures was approximately 300 μl. For the stomach samples, the sample volumes were approximately 600 μl. See Fig. 1 for feeding and sampling schedule.

### DNA extraction

DNA was extracted from all of the samples using the QIAamp PowerFaecal DNA Kit (Qiagen, Carlsbad, CA, USA). The manufacturer's recommended protocol was followed for all sample types and yielded 100 μl of a solution containing extracted DNA in a proprietary buffer named C6. These solutions were stored in 1.5 ml Eppendorf tubes and frozen at −20°C.

As a first check on the concentration of extracted DNA in the samples, 2 μl of each sample was then quantified using a Qubit 3 fluorometer and a Qubit Quantitation Assay Kits (Thermo Fisher, Scientific, Waltham, MA, USA). The manufacturer's quantification protocol for low concentration double‐stranded DNA was used to assess the concentration of the extracted sample. Samples which yielded no detectable concentration of DNA were then omitted from the rest of the analysis pipeline.

### Polymerase chain reaction (PCR)

A 16 S rRNA region corresponding approximately to the V4–V5 regions with uniquely barcoded 515f and 926r PCR primers was amplified. The PCR reactions were set‐up for each week's samples using 25 μl reaction volumes with the following composition: 1 μl of extracted DNA with a concentration of approximately 0.5 ng/μl, 1.5 μl of a 1:1 515f:926r 10 μM primer mix, 12.5 μl of PCR water and 10 μl of HotMaster mix (VWR, Visalia, CA). The set of samples amplified each week varied in number, typically 12, and included one negative control containing 1 μl of PCR water in place of extracted DNA along with a unique forward and reverse primer allocated for each week's control sample. During the 4th week, an even and staggered mock microbial community was amplified, each with its own unique forward and reverse primer pair, as a positive control (Parada *et al*., 2015). These mock communities were generated based on, with predetermined relative microbial abundances, microbial taxa commonly found in the waters of the San Pedro channel. The expected and experimentally determined values for the relative abundance of each mock community member were then used to determine an average sequencing error rate. This analysis was done using MOTHUR (Schloss *et al*., 2009). The per‐base pair error rate used was 4.571e‐4.

The PCR cycling protocol was as follows: 120 s initial denaturation at 95°C, then 30 cycles: 45 s denaturation at 95°C, 45 s annealing at 50°C, 90 s extension at 68°C, 5 min final extension at 68°C.

Another check of DNA concentration was then made, this time of the PCR product, using the same protocol as the raw DNA extraction samples. PCR products which did not have a concentration of at least 1 ng/μl were regenerated from the original extracted sample material. For PCR product samples with a sufficient concentration of DNA, 2 μl of reaction product was mixed with 3 μl of SYBR Safe DNA Gel Stain (Thermo Fisher Scientific) and was analysed immediately on a 1.0% agarose gel. To check if the amplicon length matched the expected amplicon length, the samples were separated with one well‐containing 5 μl of O'GeneRulerTM 100 bp Plus DNA Ladder (Thermo Fisher Scientific). As a negative control, each week's sample set was separated with one well‐containing 2 μl of PCR water and 3 μl of SYBR Safe DNA Gel Stain. The separation voltage gradient was set at 100 V per 10 cm and run for 60 min.

All samples were cleaned and diluted to a uniform concentration of 1 ng/μl of DNA in a solution of TE using a DNA Clean & Concentrator‐25 kit (Zymo Research). A pooled sample containing 5 μl of each cleaned sample was then made, and DNA fragments with a length under 200 bp were removed using an Agencourt AMPure XP bead cleaning kit (Beckman‐Coulter, Brea, CA). This pooled sample was then cleaned and concentrated into a solution of TE at a concentration of approximately 10 ng/μl before being sequenced.

### Sequencing

The pooled sample was sequenced using 150 bp pair‐end Illumina sequencing on the MiSeq platform at Laragen (Culver City, CA, USA). All Illumina sequencing data are available at NCBI under the BioProject PRJNA416146.

### Sequence quality control and preprocessing

All sequencing libraries were processed together. Quality control, OTU clustering and taxonomy assignment were performed in MOTHUR (Schloss *et al*., 2009), using the MOTHUR MiSeq SOP (Kozich *et al*., 2013). Only overlapping regions of the contigs and removed any sequences with ambiguous bases and/or homopolymers of with lengths of at least 8 bp were retained to ensure good quality and reduce the number of spurious OTUs. To further reduce the number of spurious OTUs, singletons (i.e. OTUs containing a single read) were also removed for any downstream analysis. The sequences were aligned to SILVA 128 reference alignment (Quast *et al*., 2013) cut to the 515f to 926r region (Walters *et al*., 2015). Taxonomy was assigned with 80% confidence cut‐off, using the Silva v128 taxonomy (Yilmaz *et al*., 2014) in conjunction with the Naïve Bayesian Classifier (Wang *et al*., 2007) used in MOTHUR. Single‐linkage pre‐clustering was performed with a cut‐off of two allowed differences (Huse *et al*., 2010). Chimeras were then removed, and the remaining sequences used to create 97% OTUs using the average‐linkage clustering method.

As the length of the sequenced reads was typically 300 bp, out of an expected amplicon length of 337 bp, the resulting sequences could only be consistently determined down to the level of genus.

### Statistical analysis

The primary statistical tools used in this project were the Phyloseq (McMurdie and Holmes, 2013) and ‘Vegan’ R packages (Oksanen *et al*., 2013), with MOTHUR generated data as the input. To compare β‐diversity measures between samples, the raw sequence count data were converted to relative sequence abundances where the scaling factor was the total number of sequences per sample.

The β‐diversity measures used in this study were the Bray–Curtis distances and weighted UniFrac distances (Hamady *et al*., 2010), both generated using the Phyloseq package. The results were further analysed by MDS, implemented by the *ordinate* function in Phyloseq, and Permanova using 10 000 permutations, implemented in the *adonis* function in the Vegan package. The significance tests using the Weighted Unifrac distance values yielded similar results to those using the Bray–Curtis dissimilarity and so only the results using the Bray–Curtis dissimilarity were displayed.

Multidimensional scaling plots were colour coded by various experimental variables, such as the type of algal feed used. The visual clarity of how samples were clustering was enhanced using the *stat_ellipse* function in ggplot2 R package, with a bounding ellipse drawn at the 95% confidence interval. It should be noted that in groups with less than four samples no unique ellipse could be drawn.

The most abundant OTU bar plots were generated using the *plot_bar* function in Phyloseq. The source data for these plots were taken as the relative abundance of the 10 most abundant OTUs per month across all samples.

Analysis of potentially significant co‐occurrences between the relative abundance of common OTUs was carried out using eLSA (Xia *et al*., 2011). Only OTUs with a relative abundance > 1% were used, to manage the data set's complexity, across all time points using a replicate value of five for control group samples and 10 for the treatment group samples. Only OTU co‐occurrences, defined both for Spearman and Pearson correlations, with a false discovery rate of under 0.05 were designated as significant (See Table S2).

## Conflict of interest

None declared.

## Supporting information


**Fig. S1.** MDS plot, using the weighted Unifrac distance between samples from all 12 weeks, showing the differences between algal microbiomes between all three algal cultures.
**Fig. S2.** MDS plot using the weighted Unifrac distance between all faecal samples.
**Fig. S3.** MDS plot using the weighted Unifrac distance between faecal samples from the control group of oysters as well as the *Tetraselmis* algal microbiomes.
**Fig. S4.** MDS plot using the weighted Unifrac distance between faecal samples from the treatment group of oysters as well as all of the algal microbiomes.
**Fig. S5.** MDS plot using the weighted Unifrac distance between control and treatment group faecal samples.
**Fig. S6.** MDS plot, using the Bray–Curtis dissimilarity between samples from all 12 weeks, showing the differences between algal microbiomes between all three algal cultures.
**Fig. S7.** MDS plot using the Bray–Curtis dissimilarity between all faecal samples.
**Fig. S8.** MDS plot using the Bray–Curtis dissimilarity between faecal samples from the control group of oysters as well as the *Tetraselmis* algal microbiomes.
**Fig. S9.** MDS plot using the Bray–Curtis dissimilarity between faecal samples from the treatment group of oysters as well as all of the algal microbiomes.
**Fig. S10.** MDS plot using the Bray–Curtis dissimilarity between control and treatment group faecal samples.

**Table S1**. The read depth of all samples. Each sample is labelled with its unique forward and reverse primer pair ID, sample type, week the sample was taken, and the feed used in generating the sample.

**Table S2**. Significant co‐occurrences between the relative abundance of common OTUs carried out using eLSA. OTUs with a relative abundance greater than 1% were used. Replicate values of 5 for control group samples and 10 for the treatment group samples were used. Only OTU co‐occurrences, defined both for Spearman and Pearson correlations, with a false discovery rate of under 0.05 were designated as significant.
