# Supplementary material for: High turnover of faecal microbiome from algal feedstock experimental manipulations in the Pacific oyster (Crassostrea gigas)
Source: Microb Biotechnol. 2018 May 10;11(5):848–58. doi: 10.1111/1751-7915.13277 (PMC6116748; doi:10.1111/1751-7915.13277)
Supplement: Supplementary file 1 — Fig. S1. MDS plot, using the weighted Unifrac distance between samples from all 12 weeks, showing the differences between algal microbiomes between all three algal cultures. Fig. S2. MDS plot using the weighted Unifrac distance between all faecal samples. Fig. S3. MDS plot using the weighted Unifrac distance between faecal samples from the control group of oysters as well as the Tetraselmis algal microbiomes. Fig. S4. MDS plot using the weighted Unifrac distance between faecal samples from the treatment group of oysters as well as all of the algal microbiomes. Fig. S5. MDS plot using the weighted Unifrac distance between control and treatment group faecal samples. Fig. S6. MDS plot, using the Bray–Curtis dissimilarity between samples from all 12 weeks, showing the differences between algal microbiomes between all three algal cultures. Fig. S7. MDS plot using the Bray–Curtis dissimilarity between all faecal samples. Fig. S8. MDS plot using the Bray–Curtis dissimilarity between faecal samples from the control group of oysters as well as the Tetraselmis algal microbiomes. Fig. S9. MDS plot using the Bray–Curtis dissimilarity between faecal samples from the treatment group of oysters as well as all of the algal microbiomes. Fig. S10. MDS plot using the Bray–Curtis dissimilarity between control and treatment group faecal samples. [file MBT2-11-848-s001.docx]

Supplementary figures

|  |
| --- |
| Figure S1: MDS plot, using the weighted Unifrac distance between samples from all 12 weeks, showing the differences between algal microbiomes between all three algal cultures. Samples are colored by algal culture (CHAE = *Chaetoceros*, ISO = *Isochrysis*, and TET = *Tetraselmis*).  Significance of algal culture in differentiating algal microbiomes (F(2,14)=2.6954, p=0.0045).  Significance of weekly sampling basis time in differentiating algal microbiomes (F(1,15)=0.99099, p=0.4205)  Note: Groups with less than four samples have too few points for a unique ellipse to be drawn using the stat_ellipse function in Phyloseq. |

|  |
| --- |
| Figure S2: MDS plot using the weighted Unifrac distance between all fecal samples.  Differences are shown between fecal microbiomes based on their algal feed. Samples are colored by algal culture (CHAE = *Chaetoceros*, ISO = *Isochrysis*, and TET = *Tetraselmis*).  Significance of algal culture in differentiating fecal microbiomes (F(2,99)=9.7561, p<1e-4). |

|  |
| --- |
| Figure S3: MDS plot using the weighted Unifrac distance between fecal samples from the control group of oysters as well as the *Tetraselmis* algal microbiomes.  CON_FECAL_MONTH_1 = Fecal samples from control group weeks 1 to 4, CON_FECAL_MONTH_2 = Fecal samples from control group weeks 5 to 8, CON_FECAL_MONTH_3 = Fecal samples from control group weeks 9 to 11.  TET_MONTH_1 = Bacterial communities in *Tetraselmis* feed weeks 1 to 4, TET_MONTH_2 = Bacterial communities in *Tetraselmis* feed weeks 5 to 8, TET_MONTH_3 = Bacterial communities in *Tetraselmis* feed weeks 9 to 11. This figure demonstrates consistency in control fecal microbiomes and control dietary microbiomes.  Significance weekly sampling basis time in differentiating all fecal samples from the control group (F(1,37)=2.8668, p=0.0172).  Significance of monthly sampling basis time, phase, in differentiating all fecal samples from the control group (F(1,37)=3.6169, p=0.0046).  Significance of weekly sampling basis time in differentiating all Tetraselmis microbiomes (F(1,9)=1.3278, p=0.2514).  Significance of monthly sampling basis time, phase, in differentiating all Tetraselmis microbiomes (F(1,9)=1.7322, p=0.09699).  Significance of sample type, fecal versus algal microbiome, in differentiating fecal samples from the control group from the Tetraselmis microbiomes (F(1,48)=9.5276, p<1e-4).  Note: Groups with less than four samples have too few points for a unique ellipse to be drawn using the stat_ellipse function in Phyloseq. |

|  |
| --- |
| Figure S4: MDS plot using the weighted Unifrac distance between fecal samples from the treatment group of oysters as well as all of the algal microbiomes.  EXP_FECAL_MONTH_1 = Fecal samples from treatment group weeks 1 to 4, EXP_FECAL_MONTH_2 = Fecal samples from treatment group weeks 5 to 8, EXP_FECAL_MONTH_3 = Fecal samples from treatment group weeks 9 to 11.  TET_MONTH_1 = Bacterial communities in *Tetraselmis* feed weeks 1 to 4, ISO_MONTH_2 = Bacterial communities in *Isochrysis* feed weeks 5 to 8, CHAE_MONTH_3 = Bacterial communities in *Chaetoceros* feed weeks 9 to 11. Notice consistent cluster migration across feed types for each experimental group, corresponding to dietary regime change.  Significance of sample type, fecal versus algal microbiome, in differentiating fecal samples from the control group from the algal microbiomes (F(1,71)=4.7314, p<1e-4).  Significance of monthly sampling basis time, phase, in differentiating the algal microbiomes (F(1,8)=4.1009, p=0.0031).  Significance of monthly sampling basis time, phase, in differentiating the fecal microbiomes (F(1,61)= 11.566, p<1e-4).  Note: Groups with less than four samples have too few points for a unique ellipse to be drawn using the stat_ellipse function in Phyloseq. |

|  |
| --- |
| Figure S5: MDS plot using the weighted Unifrac distance between control and treatment group fecal samples.  CON_FECAL_MONTH_1 = Fecal samples from control group weeks 1 to 4, CON_FECAL_MONTH_2 = Fecal samples from control group weeks 5 to 8, CON_FECAL_MONTH_3 = Fecal samples from control group weeks 9 to 11.  EXP_FECAL_MONTH_1 = Fecal samples from treatment group weeks 1 to 4,_FECAL_MONTH_2 = Fecal samples from treatment group weeks 5 to 8, EXP_FECAL_MONTH_3 = Fecal samples from treatment group weeks 9 to 11.  The significance of the differences between fecal samples taken from control and treatment groups of oysters during phase 1 (F(1,41)=1.0247, p=0.3666).  The significance of the differences between fecal samples taken from control and treatment groups of oysters during phase 2 (F(1,35)= 6.3725, p<1e-4).  The significance of the differences between fecal samples taken from control and treatment groups of oysters during phase 3 (F(1,20)= 6.7674, p<1e-4). |

|  |
| --- |
| Figure S6: MDS plot, using the Bray-Curtis dissimilarity between samples from all 12 weeks, showing the differences between algal microbiomes between all three algal cultures. Samples are colored by algal culture (CHAE = *Chaetoceros*, ISO = *Isochrysis*, and TET = *Tetraselmis*).  Note: Groups with less than four samples have too few points for a unique ellipse to be drawn using the stat_ellipse function in Phyloseq. |

|  |
| --- |
| Figure S7: MDS plot using the Bray-Curtis dissimilarity between all fecal samples.  Differences are shown between fecal microbiomes based on their algal feed. Samples are colored by algal culture (CHAE = *Chaetoceros*, ISO = *Isochrysis*, and TET = *Tetraselmis*). |

|  |
| --- |
| Figure S8: MDS plot using the Bray-Curtis dissimilarity between fecal samples from the control group of oysters as well as the *Tetraselmis* algal microbiomes.  CON_FECAL_MONTH_1 = Fecal samples from control group weeks 1 to 4, CON_FECAL_MONTH_2 = Fecal samples from control group weeks 5 to 8, CON_FECAL_MONTH_3 = Fecal samples from control group weeks 9 to 11.  TET_MONTH_1 = Bacterial communities in *Tetraselmis* feed weeks 1 to 4, TET_MONTH_2 = Bacterial communities in *Tetraselmis* feed weeks 5 to 8, TET_MONTH_3 = Bacterial communities in *Tetraselmis* feed weeks 9 to 11. This figure demonstrates consistency in control fecal microbiomes and control dietary microbiomes.  Note: Groups with less than four samples have too few points for a unique ellipse to be drawn using the stat_ellipse function in Phyloseq. |

|  |
| --- |
| Figure S9: MDS plot using the Bray-Curtis dissimilarity between fecal samples from the treatment group of oysters as well as all of the algal microbiomes.  EXP_FECAL_MONTH_1 = Fecal samples from treatment group weeks 1 to 4, EXP_FECAL_MONTH_2 = Fecal samples from treatment group weeks 5 to 8, EXP_FECAL_MONTH_3 = Fecal samples from treatment group weeks 9 to 11.  TET_MONTH_1 = Bacterial communities in *Tetraselmis* feed weeks 1 to 4, ISO_MONTH_2 = Bacterial communities in *Isochrysis* feed weeks 5 to 8, CHAE_MONTH_3 = Bacterial communities in *Chaetoceros* feed weeks 9 to 11. Notice consistent cluster migration across feed types for each experimental group, corresponding to dietary regime change.  Note: Groups with less than four samples have too few points for a unique ellipse to be drawn using the stat_ellipse function in Phyloseq. |

|  |
| --- |
| Figure S10: MDS plot using the Bray-Curtis dissimilarities between samples.  CON_FECAL_MONTH_1 = Fecal samples from control group weeks 1 to 4, CON_FECAL_MONTH_2 = Fecal samples from control group weeks 5 to 8, CON_FECAL_MONTH_3 = Fecal samples from control group weeks 9 to 11.  EXP_FECAL_MONTH_1 = Fecal samples from treatment group weeks 1 to 4,_FECAL_MONTH_2 = Fecal samples from treatment group weeks 5 to 8, EXP_FECAL_MONTH_3 = Fecal samples from treatment group weeks 9 to 11. |
